# Understanding patients' mobility for treatment seeking in India

**DOI:** 10.1038/s41598-023-50184-3

**Published:** 2024-01-22

**Authors:** Ranjan Karmakar, Umenthala Srikanth Reddy, Ram Babu Bhagat

**Affiliations:** 1 Department of Migration and Urban Studies, International Institute for Population Sciences (IIPS), Mumbai, India; 2 International Institute for Population Sciences (IIPS), Mumbai, India

**Keywords:** Diseases, Health care, Health occupations

## Abstract

Healthcare systems worldwide are grappling with the challenge of providing high-quality healthcare in the face of evolving disease patterns. India, like many other countries, faces a significant treatment gap for various curable impairments, non-communicable diseases (NCDs), and cardiovascular diseases (CVDs). To address their healthcare needs, individuals often relocate in search of better treatment options. However, no studies were conducted to understand the spatial mobility. This paper explores the determinants of spatial mobility for treatment in India using data from NSS 75th round (2017–2018). A total of 64,779 individual medical cases of different diseases were taken into consideration for our analysis. Fixed effect and multinomial regression models were used to understand diseases specific mobility for treatment. It was found that those with CVDs, NCDs, and disabilities are more prone to travel outside their district for medical care. Rural and economically disadvantaged individuals also tend to travel further for treatment. The key factors impacting treatment-seeking mobility include insurance coverage, hospital quality, cost of medicine, and cost of X-rays/surgeries. The study highlights the need for improved policies to address the gap between healthcare needs and infrastructure in India, with a focus on prioritizing the development of local healthcare facilities for disabilities, NCDs, and CVDs.

## Introduction

Low and middle-income countries worldwide are experiencing rapid transition from communicable to non-communicable diseases (NCDs) and injuries. However, the health systems in developing countries exhibit disparities in addressing the burden of diseases and are challenged in delivering quality healthcare services to combat the disease burden^1^. India and other nations report a sizable unmet demand or treatment gap for various curable impairments, NCDs, and cardiovascular diseases (CVDs)^2–5^. Multiple studies have identified a significant gap in the availability of healthcare services for various communicable and non-communicable diseases in several regions of the country^1,6–9^. It is irrefutable that these countries are in dire need of effective and well-functioning healthcare systems to address the rise in the burden of diseases.

To meet their healthcare needs, people tend to move from one place to another in search of better treatment. Such spatial movement of individuals is referred to as patients’ mobility. Studies have shown that patients’ mobility to long distances can result in delayed treatment, decreased quality of care, and increased financial burden on households^10^. There is a shortfall of 79.9% specialists at the community health centres (CHCs) compared to the requirement for existing CHCs in rural areas^11^. Even if facilities are available, the quality of services is questionable. For example, studies^7,12^ found a lack of access to basic cancer services such as radiotherapy and unequal distribution of radiotherapy units across states in public sector hospitals, leading to insufficient treatment for patients. People’s preference for the private sector over the public is often due to the non-availability of facilities at government hospitals. However, better quality services in government health facilities attract a larger share of patients, irrespective of the nature of the disease, as observed in Maharashtra^13^.

In areas with higher availability and accessibility to healthcare facilities, a greater proportion of people prefer medical treatment compared to resource-poor areas ^13^. To tackle the situation, the Government of India launched various schemes and programmes to achieve the sustainable development goals (SDGs) Goal 3 and target 3.8. However, there is still an enormous gap between the burden of diseases and undertaken intervention vis-à-vis priorities in resource allocation across states. This gap manifested as the ineffective attention on curative treatment is compelling people to seek treatment from far off places.

To explain the decision-making behaviour in healthcare, various models have been developed. Distance decay and access framework are the most widely used amongst them^14–18^. While the first framework, portraits the geographical barrier to utilize healthcare, occurs when the service usage goes down as the distance between users and facilities increases. The access framework explores how different geographic factors like location of facilities, infrastructure, physical distance, geographic disparities in resource distribution and socio-economic accessibility influence the pattern of mobility for healthcare and health service utilization. Another widely recognized framework is Andersen's framework of healthcare utilization^14,19,20^, which discusses predisposing, enabling and need based factors. Predisposing conditions include demographic (i.e., age, gender) and socio-cultural factors (i.e., education, occupation, religion, ethnicity, attitude and value towards healthcare uses). Enabling conditions include individual wealth, health insurance coverage, and geographical factors (place of residence and accessibility). Need based factors are individual patient’s status of health and morbidity. On the other side, the Health Belief Model (HBM) focuses on individual patients' perceptions, discusses about perceived health susceptibility and severity, self-efficacy of help seeking, perceived barrier, larger perceived benefit, and individual's pattern of utilization^21,22^. These models try to explore different barriers to universal access to healthcare.

Our analysis is based on understanding of the health seeking behaviour of individual patients as discussed in the above models, suggesting that health seeking behaviour is a product of various demographic, socio-cultural and environmental factors, with this regards spatial access to medical resources and understanding such mobility for health care access might be an important but neglected dimension of health seeking behaviour.

To fulfil their medical needs and avail quality and affordable care, patients travel from one place or administrative area to another, which may take place in many forms and for numerous reasons. The term ‘patient mobility’ is often used interchangeably with ‘medical tourism’, but the former is more diverse and broader than the latter^23^. The term can be described and applied in many ways, but one common characteristic of all definitions is ‘travelling to receive healthcare’^24^. Different motivations related to healthcare services like availability, affordability, familiarity and perceived quality of health care, efficiency, and two types of sources of finance for treatment (i.e., insured and non-insured) were cited for patients' mobility in the context of cross-border medical tourism studies^25–27^. Studies have also emphasized unequal access to resources among different social groups as a potential reason for spatial mobility for treatment^28^. In most studies, the absence of specific healthcare facilities in their region is the most significant factor for patients' mobility. Although this issue is well known, it has not received substantial attention in domestic patients’ mobility. Studies have also explored India as a major transnational medical tourism destination and healthcare hub^29^, however, no studies to date have explored the association of diseases with spatial mobility at national level in India.

## Data and methods

### Data source

For our present study, we used data from Social Consumption in India: Health, part of 75th round of National Sample Survey (NSS). It is fifth in the health series of data collected by National Statistical Office (NSO) in India. The data provided covers length and breadth of States and Union territories of India. The survey interviewed 1,13,823 households spread across every district in the country. The main aim of the study is to provide basic quantitative information on health. The study includes general morbidity, ailments, extent use of health services, health spending across all the groups in each gender, State/UT combinations. This nationally representative survey included over 5,55,352 individuals across all ages and gender, spread across different States and UTs. The survey adopts a stratified multi stage sampling design to arrive at the unit of observation. The detailed methodology on sampling and State wise sampling distribution can be found in the report^30^.The schematic diagram below represents the selection of sample for the study.

### Outcome variable

The primary outcome variable of this study is the pattern of spatial mobility among patients seeking medical treatment outside their district. The NSS 75th Round collected data on the place of treatment/hospitalization for in-patient members over the past 365 days. The responses are recorded in five categories: (i) same district(rural area), (ii) same district (urban area), (iii) within the state, different district (rural area), (iv) within the state, different district (urban area), and (v) other states. In India, districts are the constituent administrative units/divisions of a State.We recorded these responses into two categories: "no mobility" (i and ii) and "any mobility" (iii to v) to assess the impact of the nature of diseases on spatial mobility for in-patient care. Additionally, the responses were categorized into three groups for multinomial logistic regression analysis.

### Explanatory variables

The nature of diseases is the primary explanatory variable in the study. The NSS 75th Round's Schedule 25.0 on Household Social Consumption: Health employed a 60-fold classification of ailments to gather information on self-reported health. These 60-fold classifications were grouped into seven broad categories and further categorized into six broad categories for our analysis, including (i) Infectious, (ii) Cardiovascular Diseases (CVDs), (iii) Non-Communicable Diseases (NCDs), (iv) Disability, (v) Injuries, and (vi) Other. Despite being a part of NCDs, CVDs were taken as a separate category to examine the impact of CVDs on spatial mobility for treatment, as they have a higher prevalence and require specific health facilities that may not be readily available.

### Other covariates

Other covariates such as insurance, type of hospital, duration of stay, cost of medicine and procedures, and individuals' socioeconomic characteristics such as social group, religion, monthly per-capita consumption expenditure, age, place of residence, gender, marital status, and education levels may also play a significant role in spatial mobility for treatment.

### Analytical approach

We employed two methods to evaluate the impact of disease on mobility beyond the district. The first method involved the utilization of fixed effect regression in analyzing the effect of disease on inter-district mobility. This approach aims to shed light on the impact of disease on the mobility of individuals within the same district, assuming that individuals residing in the same district have access to similar health infrastructure, transportation facilities, and local developments. The district-level fixed effect regression was utilized to control all district-level factors that may influence a respondent but are unobserved. The mathematical representation of the equation is as follows:

1 $${\text{Y}}_{{{\text{ij}}}} = \beta .{\text{X}}_{{{\text{ij}}}} + {\text{u}}_{{\text{j}}} + \varepsilon_{{{\text{ij}}}}$$

where Y_ij_ is the mobility in and out of the district, X_ij_ represents the control variables observed in the study, u_j_ represents the controls for unobserved district and individual characteristics, and ε_ij_ represents the error term. i represents individual in region j.

In the above regression analysis, the model identifies whether there was mobility, given that the individuals have lived in the same district and given the same health infrastructure, health facilities, etc. Sometimes people's choices might be influenced by their health status, affordability, and accessibility of health care.

The second method applied in this study was a multinomial logistic regression, which aimed to identify different stages of mobility among individuals while controlling for various characteristics. This analysis differentiated mobility from one district to another district or another state. The dependent variable was categorized into three categories: healthcare utilization from the same district, another district, and another state, with "no mobility" as the base outcome. The equation for the multinomial model is as follows:

2 $${\text{Pij}}={\text{Pr}}({\text{yj}}=1)=\left\{\begin{array}{c}\frac{1}{1+\sum_{k=2}^{3}{\text{exp}}({x}_{j}{\beta }_{k})} if\, i=1\\ \frac{{\text{exp}}({x}_{j }{\beta }_{i})}{1+\sum_{k=2}^{3}{\text{exp}}({x}_{j}{\beta }_{k})}, if\, i>1\end{array}\right.$$

In the above Eq. (2), x_j_ represents the set of independent variables for the *j*th observation and ${\beta }_{k}$ is the coefficient for outcome k and i represents the status of mobility.

In conclusion, the first method provided insight into the effect of disease on inter-district mobility, while the second method distinguished the stages of mobility among individuals and controlled for various factors.

## Results

### Background characteristics of patients undertaken spatial mobility

Table 1 presents the results on the influence of diseases on the spatial mobility of in-patients. The nature of the disease is having a significant impact on patient mobility. The highest mobility was observed among those suffering from impairments (24.21%), followed by non-communicable diseases (NCDs) (23.45%) and cardiovascular diseases (CVDs) (23.19%). NGO/Charity as the type of hospital was a preferred choice for mobile patients, with 29.54% opting for it. An increase in spatial mobility was observed for patients with chronic diseases who needed to stay in the hospital for longer. No significant variation was observed in spatial mobility across different income groups, but it was higher among Schedule Caste, Tribe, and OBC groups compared to others. The highest spatial mobility was observed among males (19.96%), those living in rural areas (20.61%), non-literates (19.24%), and older persons (20.71%). The effect of health insurance on patient mobility was not significant.

**Table 1:** Inpatient Characteristics of Spatial Mobility for Medical Treatment in the Last 365 Days by Disease Type, Medical Factors, and Socio-demographic Factors.

Variables		Mobility		Chi-2	
		No	Yes	Total sample	*P* value
Nature of diseases*	Infectious	88.7	11.3	9790	< 0.001
	CVDs	76.81	23.19	7685	
	NCDs	76.55	23.45	13,662	
	Disability	75.79	24.21	5832	
	Others	86.78	13.22	20,091	
	Injuries	82.04	17.96	7719	
Insurance	No	82.35	17.65	48,257	0.052
	Yes	82.78	17.22	14,081	
Type of hospital	Govt	85.97	14.03	29,721	< 0.001
	NGO/Charity	70.46	29.54	1722	
	Pvt	79.79	20.21	33,336	
Duration of stay	Up to 2 days	88.94	11.06	16,701	< 0.001
	Up to 1w	84.21	15.79	33,494	
	More than 1w	69.92	30.08	14,584	
Cost of medicine	Not received	88.24	11.76	242	< 0.001
	Free/partly free	86.47	13.53	22,633	
	On payment	79.72	20.28	41,904	
Cost of surgery	Not received	85.39	14.61	48,282	< 0.001
	Free/partly free	74.9	25.1	4929	
	On payment	71.55	28.45	11,568	
Cost of X-ray/ECG/EEG	Not received	91.22	8.78	8204	< 0.001
	Free/partly free	82.34	17.66	12,687	
	On payment	80.29	19.71	43,888	
Cost of other tests	Not received	90.25	9.75	4908	< 0.001
	Free/partly free	85.25	14.75	17,901	
	On payment	79.82	20.18	41,970	
Free advice	No	79.76	20.24	33,118	< 0.001
	yes	84.9	15.1	31,661	
Social group	ST	81.02	18.98	6,991	< 0.001
	SC	82.28	17.72	10,404	
	OBC	81.96	18.04	25,159	
	Others	83.49	16.51	19,784	
Religion	Hinduism	82.01	17.99	47,282	< 0.001
	Islam	83.75	16.25	8422	
	Christianity	87.7	12.3	4062	
	Others	81.68	18.32	2572	
Usual Monthly per cap. Cons. Exp	Poorer	83.83	16.17	8200	< 0.001
	Poor	82.97	17.03	8892	
	Middle	79.99	20.01	11,029	
	Rich	83.02	16.98	13,598	
	Richer	82.67	17.33	20,619	
Age (in years)	Up to 14	85.87	14.13	11,204	< 0.001
	15–59	82.09	17.91	39,722	
	Above 60	79.29	20.71	13,853	
Place of residence	Rural	79.39	20.61	35,982	< 0.001
	Urban	87.03	12.97	28,797	
Gender	Male	80.74	19.26	33,090	< 0.001
	Female	84.35	15.65	29,248	
Marital status	Never married	85.6	14.4	18,493	< 0.001
	Currently married	80.93	19.07	36,861	
	Widow/divorced	82.78	17.22	6984	
Levels of education	Non-literate	80.76	19.24	18,651	< 0.001
	Primary and below	84.32	15.68	16,403	
	Upper Primary	83.18	16.82	8366	
	Secondary	82.09	17.91	8238	
	HS and above	82.39	17.61	10,680	

* Pregnancy cases were excluded.

### Typologies of mobility for treatment

Table 2 presents the mobility typologies for medical treatment among in-patients over the past 365 days. The majority of patients, 82.11%, received treatment within their district, with the predominant type of mobilities being rural to urban (36.99%) and urban to urban (30.13%). A smaller proportion of patients, 14.49%, had to cross district boundaries to receive treatment within the state, with rural to urban (9.39%) being the most common type of mobility. Only 3.4% of patients had to travel outside the state for medical treatment, and most of them came from rural areas.

**Table 2:** Classification of Patient Mobility for Medical Treatment: Types and Patterns.

Typologies of spatial mobility				Percent	Frequency (N)
No mobility	82.11 (n = 52,179)	Same district	Rural-Rural	14.12	7861
			Rural–Urban	36.99	20,393
			Urban–Rural	0.87	741
			Urban-Urban	30.13	23,184
			Total	82.11	52,179
Mobility	17.89 (n = 12,600)	Another district within state	Rural-Rural	1.59	844
			Rural–Urban	9.39	5450
			Urban–Rural	0.28	201
			Urban-Urban	3.22	3373
			Total	14.49	9868
		Other state	Rural-Other state	2.28	1434
			Urban-Other state	1.12	1298
			Total	3.4	2732

**Figure 1:**
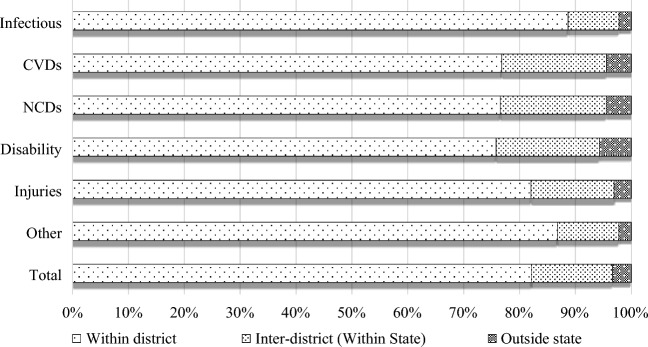
Typologies of Patient Mobility by the Nature of Diseases.

Figure 1 illustrates various typologies and rates of patient mobility based on the nature of diseases. Most patient mobility occurred within the same district for all diseases, followed by inter-district and inter-state mobility. Intra-district mobility was high across all diseases, with the highest rates observed in the treatment of infectious diseases, injuries, and other diseases. For better understading of patient mobility by different States please refer to Supplementary Figure 1A.

### Regression results

Table 3 represents the results of the fixed effect regression of the inter-district mobility of inpatients across the districts of India. In this table, we have four different specifications; the first one is the null model. In the second, overall model, we have controlled for every factor affecting inter-district mobility in India. As the literature suggests, treatment-seeking behavior differs across males and females. To capture this heterogeneous effect on treatment-seeking behavior, we have third and fourth specifications by gender.

**Table 3:** Fixed Effect Regression on Inter-district Mobility in India.

		(1)		(2)		(3)		(4)	
		Null Model	SE	Overall	SE	Male	SE	Female	SE
Nature of diseases	Other®								
	CVDs	0.118***	0.005	0.089***	0.005	0.101***	0.007	0.072***	0.008
	NCDs	0.112***	0.004	0.078***	0.004	0.087***	0.006	0.071***	0.006
	Disability	0.112***	0.006	0.089***	0.005	0.099***	0.008	0.078***	0.007
	Infectious	− 0.031***	0.005	− 0.008	0.005	− 0.003	0.007	− 0.012	0.006
	Injuries	0.041***	0.005	− 0.015**	0.005	− 0.019**	0.007	− 0.004	0.008
Insurance	No®			0	(.)	0	(.)	0	(.)
	Yes			0.010*	0.004	0.01	0.006	0.009	0.006
Type of hospital	Government®			0	(.)	0	(.)	0	(.)
	NGO/Charity			0.150***	0.01	0.128***	0.014	0.175***	0.014
	Private			0.076***	0.006	0.067***	0.009	0.085***	0.009
Duration of stay	Up to 2 days®			0	(.)	0	(.)	0	(.)
	Up to 1w			0.056***	0.004	0.058***	0.005	0.054***	0.005
	More than 1w			0.176***	0.004	0.178***	0.006	0.172***	0.006
Cost of medicine	Not received			0	(.)	0	(.)	0	(.)
	Free/partly free			− 0.064**	0.024	− 0.099**	0.034	− 0.023	0.035
	On payment			− 0.038	0.024	− 0.080*	0.034	0.011	0.035
Cost of surgery	Not received ®			0	(.)	0	(.)	0	(.)
	Free/partly free			0.094***	0.006	0.084***	0.008	0.106***	0.008
	On payment			0.073***	0.004	0.078***	0.006	0.069***	0.006
Cost of X-ray/ECG/EEG	Not received ®			0	(.)	0	(.)	0	(.)
	Free/partly free			0.035***	0.006	0.040***	0.009	0.032***	0.008
	On payment			0.022**	0.007	0.026*	0.011	0.022*	0.01
Cost of other tests	Not received ®			0	(.)	0	(.)	0	(.)
	Free/partly free			0.019**	0.007	0.013	0.01	0.022*	0.01
	On payment			0.014	0.009	0.015	0.012	0.007	0.012
Free advice	Not received ®								
	Yes			− 0.004	0.006	− 0.012	0.008	0.003	0.008
Social Group	ST								
	SC			0.015*	0.007	0.026**	0.01	0.001	0.01
	OBC			0.025***	0.007	0.041***	0.009	0.007	0.01
	Others			0.028***	0.007	0.038***	0.01	0.017	0.01
Religion	Hinduism®								
	Islam			0.003	0.005	0.007	0.007	− 0.004	0.007
	Christianity			− 0.009	0.009	0.002	0.013	− 0.017	0.012
	Others			0.019*	0.009	0.041**	0.013	− 0.003	0.013
Usual Monthly per cap. Cons. Exp				0.006***	0.001	0.006***	0.002	0.005**	0.002
Age (in years)				0	0	0	0.001	0	0.001
Age square (in years)				0	0	0	0	0	0
Place of residence	Rural®								
	Urban			− 0.020***	0.003	− 0.025***	0.005	− 0.014**	0.005
Gender	Female								
	Male			0.017***	0.003				
Marital status	Never married®								
	Currently married			− 0.002	0.006	0.01	0.009	− 0.011	0.01
	Widow/divorced			− 0.022**	0.008	− 0.016	0.013	− 0.030**	0.012
Levels of education	Non-literate®								
	Primary & below			0.003	0.004	0.003	0.006	0.002	0.006
	Upper primary			0.011*	0.005	0.014*	0.007	0.005	0.007
	Secondary			0.017***	0.005	0.017*	0.007	0.015*	0.007
	HS and above			0.024***	0.005	0.033***	0.007	0.011	0.007
	r2	0.153		0.21		0.22		0.224	
	N	64,779		62,338		33,089		29,248	

SE, Standard errors; ^squared value; ®-reference category.

Source: Authors calculation from NSS 75th round, 2017–18.

*p < 0.05, ** *p* < 0.01, *** *p* < 0.001.

The dependent variable, as defined earlier, is ‘moving out of the district for treatment seeking’. In the null model, we found that persons with CVDs, NCDs, disabilities and injuries are more likely to migrate away from the district than persons suffering from other diseases. About 11.8 percent of the population suffering from CVDs, equally 11.2 percent from NCDs, and disabilities tend to move out of the district for treatment. At the same time, people suffering from the infectious disease do not tend to move out of the district.

In the overall model (column 2), after controlling for socioeconomic and demographic factors, we found that people suffering from CVDs (8.9 percent), NCDs (7.8 percent), and disabilities (8.9 percent) tended to move out of the district. After controlling for several factors, we found that people suffering from injuries do not move away from the district compared to people suffering from other diseases. As compared to individuals with no insurance, individuals who are insured are 10 percent more likely to move out from the district for treatment of the diseases. Individuals who availed treatment from NGO/Charity and private hosiptals are more likely to move away from their districts as compared to people who availed treatment from government facilties. Factors like the cost of medicine, x-ray, and surgery also tend to influence mobility when provided free/partly free. Among the social groups as compared to scheduled tribes, the scheduled caste, OBC and others are more likely to move away from their district for health care facility. With the increase in monthly per capita expenditure, there is an increase in mobility out of the district. People living in rural areas are about 2 percent more likely to move out of the district for treatment than the urban residents. With an increase in education, there is a rise in mobility trends from their respective districts.

In the third and fourth specifications, we have shown the heterogeneous effect of availing treatment services when suffering from a disease on inter-district mobility. For males and females, we found similar results of inter-district mobility for treatment seeking. Males, when infected with CVDs, NCDs, and disabilities, are about 10 percent more likely to move out of the district than when suffering from other diseases. On the other hand, females suffering from NCDs, CVDs, and disabilities are about 7 percent more likely to move out of the district.

Table 4 represents the analysis of heterogeneous effects on spatial (inter-district) mobility when suffering from a disease in India. The literature suggests that each age has different health care requirements and availing of health services also differs as per the place of residence. To account for such heterogeneity, we have considered four specifications. The first pertains to the child (0–14 years) specification, the second is the young (15–59 years) specification, the third specification is for older adults (60 + years), fourth and fifth specifications are for rural and urban residents. Each model yields consistent results. In each specification, we found that people suffering from CVDs, NCDs, and disability tended to move out of their district for treatment-seeking while people suffering from injuries and infectious diseases are less likely to move out from their respective districts. In child specification, we found that 16 percent of them have moved out of their home district for CVDs treatment while 8.8 percent of them have moved out for NCDs treatment and 14 percent for disability treatment. When young adults suffered from a CVDs disease, 9 percent are more likely to moved outside their district, while 7 percent moved outside when they suffered from NCDs. In rural areas, too people have higher chances to move outside the district when infected with CVDs and NCDs (9 percent and 8.2 percent) while in urban areas, the mobility is about 8.9 percent and 7 percent respectively. To capture the heterogeneity by different regions of India (Supplementary Table 1A), an analysis by regions is carried out. We found the pattern of mobility is consistent with the pattern observed in the overall model for each category of disease, however we found that across regions, the results for individuals who suffered from injuries were not significant.

**Table 4:** An Analysis of Heterogeneous Effects on Inter-District Mobility in India.

		(1)		(2)		(3)		(4)		(5)	
		Child model		Young adult model		Elderly model		Rural model		Urban model	
Nature of diseases	Other®										
	Infectious	− 0.001	(0.008)	− 0.01	(0.006)	− 0.016	(0.013)	− 0.017**	(0.006)	0.004	(0.007)
	CVDs	0.162***	(0.021)	0.092***	(0.007)	0.055***	(0.01)	0.090***	(0.008)	0.089***	(0.007)
	NCDs	0.088***	(0.01)	0.072***	(0.005)	0.064***	(0.01)	0.082***	(0.006)	0.070***	(0.006)
	Disability	0.140***	(0.015)	0.093***	(0.007)	0.038**	(0.012)	0.076***	(0.008)	0.103***	(0.008)
	Injuries	0.001	(0.013)	− 0.016*	(0.006)	− 0.029*	(0.014)	− 0.018*	(0.007)	− 0.011	(0.007)
	r2	0.20		0.22		0.287		0.222		0.245	
	N	11,001		38,693		12,624		34,646		27,691	

Note: All the models were controlled for following varibales : Insurance; Type of Hospital; Duration of Treatment/Stay; Cost of Medicine; Cost of Surgery; Cost of X-ray/ECG/EEG; Cost of Other tests; Free advice; Social group; religion; Usual monthly per captia consumption; Age; Place of residence; Gender; Marital Status; Levels of Education;

Standard errors in parentheses; ®-reference category.

Source: Authors calculation from NSS data.

*p < 0.05, ** *p* < 0.01, *** *p* < 0.001.

### Multinomial results

We carried out multinomial logistic regression to identify the mobility pattern among inter-district and inter-state health mobility, the results for which are presented in Tables 5 & 6. As there are urban–rural differentials in access to health care utilization in India, an urban and rural specification was carried along with the overall specification to observe this heterogeneity. To study the health care utilization within the same district (of residence), another district and other, state multinomial logistic regression is adopted, adjusting for variables like age, gender, sex, marital status, education, religion, caste, place of residence, nature of disease, insurance, type of medical, duration of stay, cost etc.,

**Table 5:** Multinomial Logistic Regression Analysis of the Spatial Distribution of Mobility by Place of Residence.

		Rural model				Urban model			
		Same district vs another district		Same district vs another state		Same district vs another district		Same district vs another state	
		RR	SE	RR	SE	RR	SE	RR	SE
Nature of disease	Other®								
	Infectious	0.817***	0.044	1.022	0.112	1.02	0.074	1.128	0.134
	CVDs	1.668***	0.089	2.121***	0.209	1.876***	0.12	2.113***	0.211
	NCDs	1.742***	0.073	1.789***	0.149	1.778***	0.098	1.933***	0.168
	Disability	1.601***	0.086	2.121***	0.208	2.267***	0.152	2.177***	0.235
	Injuries	0.898*	0.047	0.869	0.089	1.12	0.079	0.784	0.097
Social group	ST®	1	(.)	1	(.)	1	(.)	1	(.)
	SC	0.967	0.056	1.031	0.123	0.87	0.09	0.329***	0.052
	OBC	1.003	0.053	1.01	0.113	1.07	0.105	0.486***	0.063
	Others	0.868*	0.049	1.296*	0.148	0.896	0.087	0.534***	0.068
Religion	Hinduism®								
	Islam	1.202***	0.056	0.873	0.086	1.097	0.059	1.179	0.103
	Christianity	1.018	0.069	0.579***	0.095	0.647***	0.067	0.450***	0.075
	Others	1.012	0.075	1.327*	0.164	1.078	0.108	1.252	0.177
Age (in years)		1.010*	0.004	1.001	0	1.001	0	0.996	0
Gender	Female®								
	Male	1.106**	0.035	1.376***	0.086	1.051	0.043	1.202**	0.078
Marital Status	Never married®								
	Currently married	0.843*	0.057	1.163	0.154	1.022	0.087	1.259	0.176
	Widow/divorced	0.753**	0.066	0.961	0.167	0.841	0.093	0.863	0.158
Levels of education	Non-literate®								
	Primary & below	0.941	0.037	0.985	0.075	0.883*	0.051	0.949	0.094
	Upper primary	1.006	0.049	1.07	0.1	0.992	0.068	1.019	0.118
	Secondary	1.05	0.055	1.134	0.112	1.013	0.066	1.059	0.115
	HS and above	1.132*	0.062	1.234*	0.124	0.985	0.062	1.092	0.112
	N	34,647				27,691			

All the models were controlled for Insurance; Type of Hospital; Duration of Stay; Cost of medicine; Cost of Surgery; Cost of X-ray/ECG; Cost of other tests; Free Advices.

SE, Standard errors; ®-reference category Source: Authors calculation from NSS data * *p* < 0.05, ** *p* < 0.01, *** *p* < 0.001.

**Table 6:** Multinomial Logistic Regression Analysis of the Spatial Distribution of Mobility.

		Overall model			
		Same district vs another district		Same district vs another state	
		RR	SE	RR	SE
Nature of disease	Other®				
	Infectious	0.883**	0.038	1.063	0.085
	CVDs	1.739***	0.071	2.108***	0.147
	NCDs	1.752***	0.058	1.864***	0.112
	Disability	1.837***	0.076	2.159***	0.156
	Injuries	0.975	0.041	0.841*	0.066
Social group	ST®				
	SC	0.944	0.047	0.710***	0.066
	OBC	1.032	0.047	0.790**	0.066
	Others	0.868**	0.041	0.921	0.078
Religion	Hinduism®	1	(.)	1	(.)
	Islam	1.181***	0.041	1.036	0.067
	Christianity	0.878*	0.049	0.558***	0.064
	Others	1.03	0.061	1.310**	0.122
Age (in years)		1.002*	0	1	0
Place of residence	Rural®	1	(.)	1	(.)
	Urban	0.587***	0.015	0.763***	0.035
Gender	Male	1.087**	0.027	1.294***	0.058
	Female®	1	(.)	1	(.)
Marital Status	Never married®	1	(.)	1	(.)
	Currently married	0.907	0.048	1.201*	0.094
	Widow/divorced	0.785**	0.054	0.905	0.107
Levels of education	Non-literate®	1	(.)	1	(.)
	Primary and below	0.921*	0.029	0.961	0.05
	Upper primary	1.013	0.039	1.04	0.073
	Secondary	1.045	0.041	1.082	0.075
	HS and above	1.04	0.04	1.140*	0.075
	N	62,338			

All the models were controlled for Insurance; Type of Hospital; Duration of Stay; Cost of medicine; Cost of Surgery; Cost of X-ray/ECG; Cost of other tests; Free Advices.

SE, Standard errors; ®-reference category Source: Authors calculation from NSS data * *p* < 0.05, ** *p* < 0.01, *** *p* < 0.001.

From Table 6 (overall model) it can be observed that compared to the people suffering from other diseases, people suffering from CVDs, NCDs and disability are more likely to move out from their district to another district and other state. The mobility pattern has been consistent for people suffering in rural and urban areas (Table 5). People with insurance are less likely to move out from their district than those without insurance. For treatment seeking it is more likely to go to a private and charity hospital while moving out from their home district to another district and other states. As compared to scheduled tribes others are less likely to move to another district overall and especially in rural areas. As compared to scheduled tribes, scheduled castes and OBCs are less likely to move to other states for treatment seeking. It is found that people from rural areas in other social groups have higher chances of moving out from their district to other states (RR: 1.2). In overall, by religious groups Muslims are more likely to move to another district for health care (RR: 1.18) and in rural areas (RR: 1.2) as compared to Hindus. Christians are less likely to move to either another district or to another state as compared to Hindus. Compared to females, males are more likely to move to another district and another state for treatment seeking. In rural areas compared to females, males are more likely to have inter-state mobility (RR: 1.3) and to another district (RR: 1.1). The model didn’t observe any significant pattern of mobility by education level. In the overall model, currently-married people are more likely to to move out of state (RR: 1.2) than to be in their district for health care compared to never-married persons, while widowed/divorced people less likely to move to another district (RR: 0.7).

## Discussion

The current study focuses on how different sections of people driven by unmet needs move outside their usual residence to other areas to avail treatment. With the increasing burden of diseases in India, the demand for healthcare facilities has increased many folds, however, the improvements in the health facilities are not adequate when compared with the increasing demand. The analysis of spatial mobility for treatment in India has the following salient features. In all the models, we found that people suffering from CVDs, NCDs, and disabilities are more likely to move out from their native place to other places for treatment seeking as compared to people suffering from other diseases. People who suffered from infectious diseases and injuries are less likey to move out of their home district for health care seeking. Secondly, the stream of mobility within the district is more from rural to urban for disease treatments in India. Third, people (from their home district) are more likely to move to another district for treatment seeking than moving to another state for treatment seeking.

Various studies across the world have shown the degree of care gap amongst various groups^2–4^ but have not explored the scenario of spatial mobilities emerged out of the unmet need. In these circumstances, the current study with strong empirical evidence shows the impact of the nature of diseases on the geographical mobility of patients and the various spatial typologies. The study provides much-needed insight that the unmet need for curative care services (depending on the nature of diseases) and the spatial inequality in health care services push people to move outside their areas for treatment. Moreover, the change in epidemiological transition has to be considered as an important factor while strengthening any healthcare system to provide universal health coverage for all inpatient care, which is also corroborated by other researchers^13^.

Spatial mobility for treatment manifests the burden of diseases and the regional imbalance in related healthcare facilities. In India, it is found that almost one-fifth of the patients had travelled to some other places out of their place of residence. The study tried to capture the various typologies of treatment-related mobilities across districts and states and found that more than eighty percent of the patients seek treatment within their home district. However, within the district mobilities had mostly taken place in-between rural–urban places (39.0%) followed by urban-urban places (30.0%), reflecting the unmet need at local levels within the district and variations in healthcare facilities also exist in the case of each district especially in rural areas. Again, 14.5 percent of people undertake inter-district mobility, most of which is seen to happen from rural to urban areas (9.39%), followed by a narrow stream of urban-urban mobility (3.22%). In addition, 3.4 percent of the patients travelled inter-state for treatment, out of which 2.28 percent travelled to other states from rural areas. Thus, it can be concluded that mobility from rural areas to some nearby or far urban places to get treatment for curative diseases is most prevalent. Similar experiences were reported by various studies^10,31,32^ where lack of health facilities was one of the major reasons people from rural areas moved for better treatment to urban places. Thus, a stream of mobility from rural to urban might also lead to developing health regions with concentrated regions of health facilities^33^.

In the case of diseases, it is interesting to find that intra and inter-district mobility is different for different diseases. Patients suffering from injuries and infectious diseases mostly prefer to treat themselves within the district. A possible explanation may be that infectious diseases restrict spatial mobility, whereas accidents and injuries require the immediate attention of a medical practitioner, and are thus treated locally. Patients suffering from chronic diseases like NCDs, CVDs and disability may choose to go to other places based on their choice and perception regarding the quality and availability of healthcare services in destination areas. It is also interesting to note that disabilities patients from urban areas have higher chances of moving out to some other district or state due to higher accessibility.

Various facility-level factors like the type of hospitals, availability of free medicine, X-ray/ECG/EEG, Surgery, and insurance was considered, all of it has significance in influencing the decision to move outside the district especially when provided free. People preferred to go to some NGO/Charity or private hospital facilities more than government hospitals in general which is also corroborated in earlier studies^13^. Irrespective of their socio-demographic differences people have moved from one place to another place for treatment of NCDs, CVDs and disabilities, which shows the unmet need and the prevailing imbalance in existing healthcare services.

Sometimes there are policies that enable the patients to choose freely among health care providers. The portability feature of the different Central and State Government sponsored insurance scheme allows beneficiaries to use their entitlements in any empanelled hospital across the nation^34^. Moreover, the referral system also generates patients flow towards tertiary care hospitals in India^35^. These policies aim to create competition, increase productivity, and improve the quality of medical care, also play important role in equalization of the public health services through increasing footfall of migrant patients^36^. However, in this regard, further studies are necessary in Indian perspective.

Although this study has several important findings that haven't been explored in the Indian setting, it still has certain limitations that need to be examined in more detail. Firstly, information on the place and details of hospitalization and morbidity episodes were self-reported. The survey dataset does not provide information on specific healthcare facilities or their temporal characteristics at the state or district level. Moreover, the survey does not provide any information on the exact location of hospital visited by individual patients, their visiting time and distance travelled. We have also excluded the cases related to institutional delivery due to their differences with other diseases and the nature of medical treatments sought during pregnancy. The respondents self-reported all the information regarding the cost of medical care and sources of financial assistance and may suffer from recall bias. Finally, the study is based on cross-sectional data, which provides no causal relationship between the processes.

### Supplementary Information

Supplementary Table 1.

Supplementary Figure 1.

## Data Availability

We have used secondary data for the Analaysis. The data set is in public domain. One can access the data from the following link. Home (microdata.gov.in).
