# Supplementary material for: Understanding patients' mobility for treatment seeking in India
Source: Sci Rep. 2024 Jan 22;14:1887. doi: 10.1038/s41598-023-50184-3 (PMC10803797; doi:10.1038/s41598-023-50184-3)
Supplement: Supplementary file 1 — Supplementary Table 1. [file 41598_2023_50184_MOESM1_ESM.docx]

**Supplementary Tables**

**Table 1A:** **Fixed effect regression on intra-district mobility by different regions in India.**

| **Spatial Mobility** | **North** | | **Central** | | **East** | | **West** | | **South** | | **North East** | |
| --- | --- | --- | --- | --- | --- | --- | --- | --- | --- | --- | --- | --- |
|  | **Beta (Coeff)** | **SE** | **Beta (Coeff)** | **SE** | **Beta (Coeff)** | **SE** | **Beta (Coeff)** | **SE** | **Beta (Coeff)** | **SE** | **Beta (Coeff)** | **SE** |
| Infectious | 0.014 | -0.012 | 0.014 | -0.012 | -0.026^*^ | -0.011 | 0.011 | -0.011 | -0.003 | -0.01 | -0.027^*^ | -0.011 |
| CVDs | 0.116^***^ | -0.013 | 0.118^***^ | -0.014 | 0.067^***^ | -0.014 | 0.064^***^ | -0.011 | 0.100^***^ | -0.01 | 0.073^***^ | -0.016 |
| NCDs | 0.099^***^ | -0.011 | 0.084^***^ | -0.011 | 0.051^***^ | -0.01 | 0.046^***^ | -0.009 | 0.097^***^ | -0.008 | 0.078^***^ | -0.012 |
| Disability | 0.128^***^ | -0.014 | 0.115^***^ | -0.014 | 0.087^***^ | -0.015 | 0.081^***^ | -0.013 | 0.076^***^ | -0.01 | 0.062^***^ | -0.016 |
| Other® |  |  |  |  |  |  |  |  |  |  |  |  |
| Injuries | 0.003 | -0.013 | -0.02 | -0.013 | -0.015 | -0.012 | -0.019 | -0.012 | -0.016 | -0.01 | 0.002 | -0.016 |
| r2 | 0.218 |  | 0.24 |  | 0.212 |  | 0.126 |  | 0.167 |  | 0.319 |  |
| N | 10978 |  | 10249 |  | 10375 |  | 7986 |  | 15609 |  | 7141 |  |

Standard errors in parentheses; All the models are controlled for all the variables as shown in Table3.

Source: Authors calculation from NSS 75^th^ round, 2017-18

^*^ *p* < 0.05, ^**^ *p* < 0.01, ^***^ *p* < 0.001
