# Supplementary figures and images for: Understanding patients' mobility for treatment seeking in India
Source: Sci Rep. 2024 Jan 22;14:1887. doi: 10.1038/s41598-023-50184-3 (PMC10803797; doi:10.1038/s41598-023-50184-3)

**Supplementary Figure**

**Figure 1A : Spatial mobility of pateints by different states in India.**


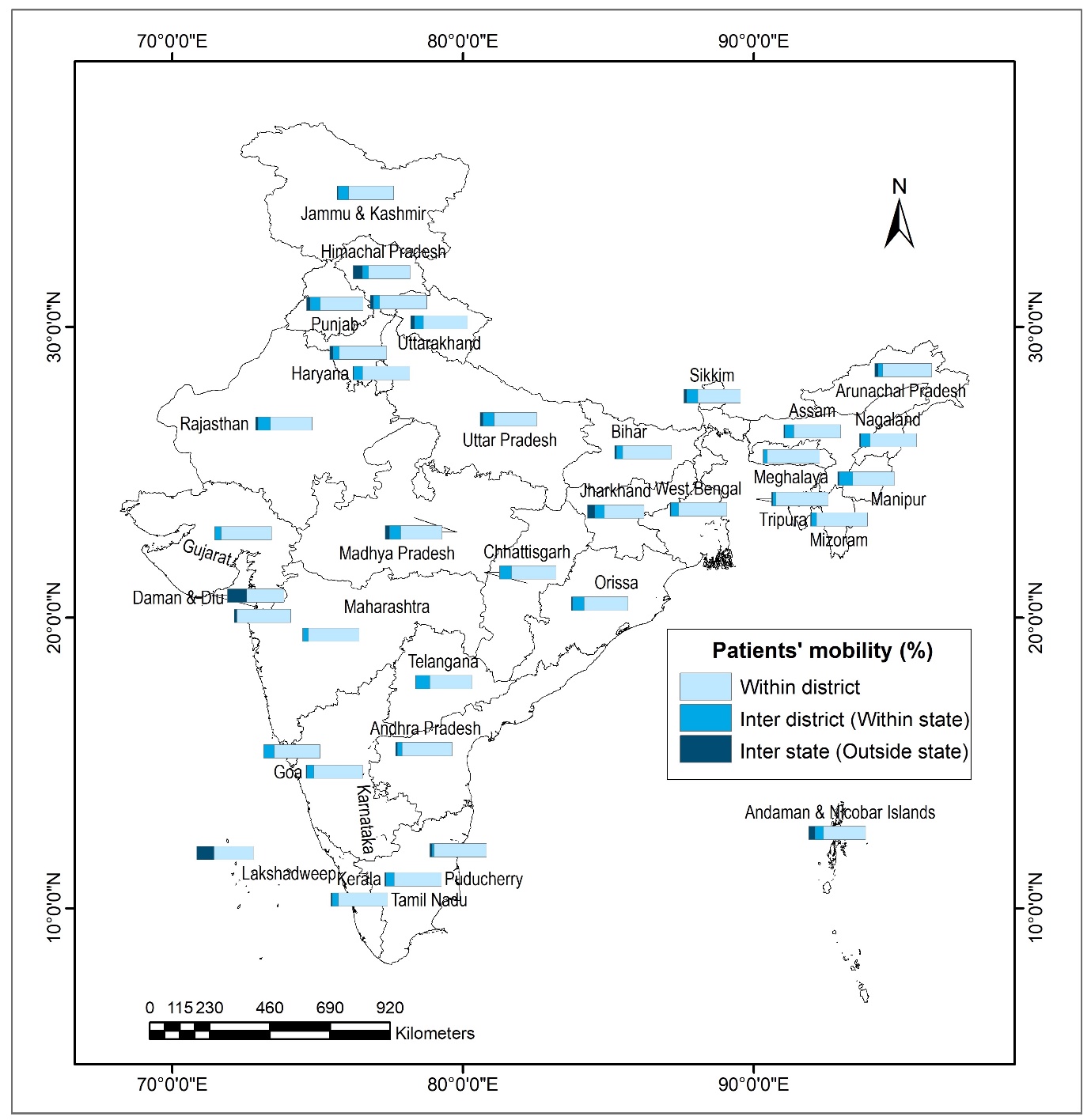

Supplement: Supplementary file 2 — Supplementary Figure 1. [file 41598_2023_50184_MOESM2_ESM.docx]
